# An Assessment of the Quality and Reliability of Gastric Botox Information Videos on YouTube

**DOI:** 10.7759/cureus.44747

**Published:** 2023-09-05

**Authors:** Suleyman Caglar Ertekin

**Affiliations:** 1 General Surgery, Private Clinic, Izmir, TUR; 2 General Surgery, Altınbas University, İstanbul, TUR

**Keywords:** discern score, jama score, youtube videos, obesity, gastric botox

## Abstract

Background and objective

Intragastric botulinum toxin (Botox) applications have emerged as a novel endoscopic intervention method to treat obesity. YouTube stands out as one of the primary online platforms frequently utilized for accessing health-related information. In light of this, this study aimed to evaluate the accuracy and reliability of informational videos about gastric Botox on YouTube.

Materials and methods

In July 2023, a comprehensive evaluation of gastric Botox information videos on YouTube was conducted by querying "Gastric Botox" on YouTube. A total of 70 videos were initially reviewed and 48 videos were meticulously analyzed by a general surgeon. Videos were categorized based on their sources and attributes and evaluated using standard scales like the Journal of the American Medical Association (JAMA) score, modified DISCERN, and the Global Quality Scale (GQS).

Results

A total of 48 videos were assessed. Of these, 2.1% originated from academic institutions, 20.8% from private hospitals/organizations, and 52.1% from physicians. Videos by other healthcare professionals accounted for 2.1%, health information websites 12.5%, and independent users 10.4%. Video durations varied significantly across sources (p<0.001). Independent user videos had the highest likes/views. JAMA scores (p=0.009) and DISCERN scores (p=0.045) showed significant differences among sources. Academic institution videos had a median JAMA score of 4; independent users scored 1. As for DISCERN, academic videos scored the highest at 5, while independent users scored the lowest at 1.8.

Conclusions

YouTube is filled with healthcare information videos today. Although the quality and reliability scores based on conventional assessment methods might be moderate, we advise utilizing videos from academic institutions and reputable health information websites as primary sources to educate patients about gastric Botox.

## Introduction

Obesity has emerged as a substantial global health concern, and its incidence has risen significantly over the past decade [1]. Consumption surpassing normal levels and heightened appetite represent paramount challenges encountered by individuals grappling with obesity. An emerging approach within the field of endoscopic interventions for obesity involves the direct administration of botulinum toxin-A (BTA) into the gastric wall. This innovative approach holds the potential to retard gastric emptying while concurrently augmenting the sensation of fullness, achieved through the transient paralysis induced at the injection site. The underlying mechanism of BTA pertains to the inhibition of acetylcholine release within cholinergic neuromuscular connections. It is noteworthy that the impact of this intervention undergoes a gradual wane within the initial three to six months and does not lead to any enduring structural detriment [2].

Currently, there has been a notable surge in the utilization of online platforms as sources of health-related information. Among these digital platforms, YouTube has gained prominence as one of the preferred mediums accessed by patients. The viewing of healthcare-related videos on YouTube has progressively become advantageous for patients in managing their medical conditions. The advent of the coronavirus disease 2019 (COVID-19) pandemic has underscored the heightened significance of social media's influence on patients, surpassing its impact in previous times [3,4].

To the best of our knowledge, there is currently a lack of research investigating the content of videos on YouTube concerning intragastric botulinum toxin (Botox) applications for obesity treatment. Therefore, the principal objective of this study was to assess the accuracy, credibility, and dependability of information conveyed on YouTube videos addressing intragastric Botox applications within the context of obesity management.

## Materials and methods

In July 2023, we commenced our study by entering a query for the term "Gastric Botox" into the search field of the YouTube platform after ensuring that the language of preference was set as English. The search was conducted by utilizing the default filters. Subsequently, the initial 70 videos that were ranked by the YouTube algorithm underwent comprehensive evaluation. After the application of the predetermined exclusion criteria, 22 videos were excluded and the remaining 48 videos were meticulously reviewed by a general surgeon who performed over 200 intragastric Botox applications annually. The assessment process involved the application of designated scoring systems to determine the videos' quality and pertinent attributes.

The exclusion criteria we employed were as follows: duplicate videos, non-English videos, restricted videos, videos featuring promotional content for hospitals or medical devices, videos primarily centered on personal experiences with illness, videos lacking relevance, videos shorter than one minute, and those devoid of auditory elements or substantive information. The provenance of the videos was classified into distinct categories based on their source, namely, academic institutions, professional organizations, physicians, healthcare professionals other than physicians (such as biologists, pharmacists, and medical students), health information websites, and independent contributors.

For the evaluation of the videos, we employed three commonly employed standard assessment scales for content evaluation. To measure video reliability, the 4-criteria Journal of the American Medical Association (JAMA) benchmarks were applied. Moreover, the Global Quality Scale (GQS) was used to evaluate the educational content quality of the videos. Additionally, the modified DISCERN score was employed to examine both the reliability and quality aspects of the videos [5-9].

An overview of the scoring systems is as follows: GQS scores range from 1 (indicating poor quality) to 5 (indicating excellent flow and quality). The JAMA score assigns 1 point for each of the specified components, namely authorship, attribution, currency, and disclosures. The modified DISCERN score consists of five questions, each answered with either a yes (1 point) or no (0 points). Higher scores indicate enhanced reliability, consistent with previous literature. The cumulative GQS, JAMA, and modified DISCERN scores for each video were determined by calculating the average of the scores provided by the assessor (Table 1).

**Table 1:** Standard scales used for the evaluation of YouTube videos GQS: Global Quality Scale; JAMA: Journal of the American Medical Association

JAMA: establishes evaluation criteria (1 point for each criterion, yielding a total score of 4 points)	GQS: assigns scores ranging from 1 (indicating poor quality) to 5 (indicating excellent flow and quality)	The modified DISCERN: employs a scoring mechanism where 1 point is attributed for each "Yes" and 0 points for each "No."
Authorship: Information regarding the credentials and affiliations of authors and contributors must be furnished	Score 1: The video exhibits low quality, lacks a coherent structure, lacks substantial information, and offers minimal utility to patients	Does the video exhibit clarity, conciseness, and comprehensibility?
Attribution: Presents copyright details comprehensively and cites references and sources for the content	Score 2: The video displays an overall subpar quality and lacks a smooth presentation. While some information is included, numerous crucial topics are absent, resulting in limited value for patients	Does the video rely on credible sources of information? (e.g., citing publications, featuring specialist speakers)
Currency: The original posting date of the content and any subsequent updates should be supplied	Score 3: The video demonstrates a moderate level of quality, although its flow could be improved. While certain important information is sufficiently addressed, other aspects are inadequately covered, leading to moderate usefulness for patients	Does the information provided maintain a balanced and impartial perspective?
Disclosure: Any potential conflicts of interest, funding sources, sponsorships, advertising affiliations, support, and ownership of the video need to be completely revealed	Score 4: The video showcases a commendable quality with a smooth flow. It effectively includes substantially pertinent information, yet some topics remain unaddressed. The video is valuable for patients	Are supplementary information sources provided for patient reference?
	Score 5: The video is characterized by exceptional quality and seamless flow, offering significant utility to patients	Are any areas of uncertainty or controversy acknowledged?

Descriptive research encompassed the examination of publicly accessible internet videos targeting a general audience. Notably, this study did not involve human or animal participants. Given the absence of patient-related data or materials and the availability of all videos for public access on the social media platform YouTube.com, institutional review board or ethics committee approval was deemed unnecessary for this investigation.

Statistical analysis

Baseline clinical data underwent appropriate statistical analyses based on the nature of the data. Continuous data were analyzed using t-tests or Mann-Whitney U tests, while categorical data underwent analysis using the Fisher's exact test or chi-square test. The statistical analysis was performed using the IBM SPSS Statistics software version 22.0 (IBM Corp., Armonk, NY). Descriptive statistical methods were employed to characterize the study data, including measures such as mean, standard deviation (SD), median, frequency, percentage, and minimum and maximum values. Comparisons of normally distributed quantitative variables between the two groups were performed using the one-way ANOVA test. It is important to note that all statistical tests followed a two-tailed approach, and a significance level of p<0.05 was utilized to establish statistical significance.

## Results

A total of 48 videos were evaluated. Of these, 2.1% of the videos were provided by academic institutions, and 20.8% were shared by private hospitals or organizations. Moreover, a significant proportion of the videos (52.1%) were created by physicians. While healthcare professionals other than physicians accounted for 2.1% of the videos, the proportion of videos by health information websites was determined to be 12.5%. Videos prepared by independent users constituted a share of 10.4%. Regarding the assessment of video characteristics, the analysis revealed that the median duration of videos was 4.27 minutes. The median number of likes for the videos was calculated to be 603, while the median number of views was determined as 29,635. Furthermore, the median duration since video upload was found to be 743 days. The evaluation of video quality was also conducted based on the assessment results. The median GQS score for the videos was 2.29, the median JAMA score was 2.18, and the median modified DISCERN score was 2.85 (Table 2).

**Table 2:** Detailed characteristics of intragastric Botox videos GQS: Global Quality Scale; JAMA: Journal of the American Medical Association

Parameters	Values (N=48)
Video source, n (%)	
Academic institutions	1 (2.1)
Private hospitals, organizations	10 (20.8)
Physicians	25 (52.1)
Healthcare professionals other than physicians	1 (2.1)
Health information websites	6 (12.5)
Independent users	5 (10.4)
Duration, minutes, median (min-max)	4.27 (1.21–30)
Likes, median (min-max)	603 (0–16,302)
Views, median (min-max)	29,635 (24–799,000)
Duration since video upload date, days, median (min-max)	743 (15–2,555)
GQS score, median (min-max)	2.29 (1–4)
JAMA score, median (min-max)	2.18 (1–4)
DISCERN score, median (min-max)	2.85 (1–5)

The duration of videos varied significantly across different sources (p<0.001). Videos from academic institutions had the longest median duration (30 minutes), while videos from healthcare professionals other than physicians had a median duration of 2.92 minutes. Videos from private hospitals or organizations, physicians, and independent users also showed statistically significant variations in duration. Likewise, the number of likes and views also demonstrated statistically significant differences among the sources (p=0.001). Videos from independent users had the highest median number of likes (5,626) and views (261,330), both of which were notably higher compared to other sources. Videos from academic institutions and healthcare professionals other than physicians had relatively lower median likes and views.

Regarding the duration since the video upload date, although there was no statistically significant difference among the sources (p=0.144), the median duration varied. Videos from academic institutions had the longest median duration since upload (577 days), while those from private hospitals or organizations had a median duration of 914 days. In terms of quality assessment scores, there were some statistically significant differences. The JAMA score had significant variations among the sources (p=0.009): videos from academic institutions had the highest median JAMA score of 4, while those from independent users had the lowest median score of 1. Furthermore, the modified DISCERN score showed significant differences among the sources (p=0.045), with videos from academic institutions having the highest median score of 5, while those from independent users had the lowest median score of 1.8 (Table 3).

**Table 3:** Assessment of video sources based on video characteristics GQS: Global Quality Scale; JAMA: Journal of the American Medical Association

Video features	Academic institutions	Private hospitals, organizations	Physicians	Healthcare professionals other than physicians	Health information websites	Independent users	P-value
Duration, minutes, median (min-max)	30	2.12 (1.01–6.42)	3.11 (0.42–13)	2	2.92 (0.43–11.08)	11.31 (0.34–26.2)	<0.001
Likes, median (min-max)	1	10 (0–45)	23 (0–122)	1	26 (2–71)	5,626 (2–16,000)	0.001
Views, median (min-max)	93	1,234 (29–5,400)	3,639 (26–35,021)	29	2,067 (24–7,721)	261,330 (149–799,003)	0.001
Duration since video upload date, days, median (min-max)	330	577 ( 60–1,901)	914 (30–2,555)	15	433 (30–1,050)	821 (370–995)	0.144
GQS score, median	3	2.3	2.56	2	1.66	1.60	0.174
JAMA score, median	4	2.2	2.4	2.01	2	1	0.009
DISCERN score, median	5	2.9	3.16	2	2.16	1.8	0.045

## Discussion

This study represents the first endeavor to assess the information pertaining to the intragastric Botox application available on the YouTube platform. Forty-eight videos, with a cumulative viewership of 1.423 million, were evaluated by a general surgery specialist experienced in the field of intragastric Botox application. Based on the videos' content, responses were sought from the patient's standpoint to the following queries: What is intragastric Botox, in what circumstances is it applied, and for which patients is it suitable? How does the post-procedural process unfold? What are the associated risks and side effects? What is the extent of its efficacy? What post-procedural guidelines should be adhered to, and what potential complications might arise?

In the context of our study, an evaluative scoring procedure was applied to the information disseminated through the videos, leading to the identification of moderate video quality. However, it was observed that videos originating from academic institutions and healthcare-oriented online platforms manifested high levels of reliability and quality. This study, which presents findings consistent with prior research on gallstone disease, thyroid surgery, and Helicobacter pylori, aligns with other scholarly investigations focused on evaluating educational content on YouTube [6,10,11].

However, there are also studies that have reached different conclusions about YouTube videos as a valuable resource for patients [3]. A meta-analysis involving 202 articles concluded that YouTube cannot be considered a reliable source of medical and health-related information. The analysis suggested that YouTube should improve its ranking and recommendation system to promote the availability of higher-quality health-related content [5]. In our study, utilizing the three most common scoring systems, we identified a positive correlation among these systems. Various aspects of video sources and features were compared within the framework of these scoring systems. Academic institutions and healthcare professionals tended to produce more reliable and high-quality videos compared to other sources [8,12,13].

Patients may often display a tendency to conduct research on social media platforms and watch YouTube content related to gastric Botox when they seek medical attention for the procedure or after undergoing an examination. In this context, it is essential for patients to exercise caution while obtaining health information from the internet and to utilize reliable sources. This study has a few limitations. Our assessments could be deemed superficial due to the absence of a validated tool for evaluating data from videos on a specific topic. Furthermore, the videos were searched based on YouTube's default settings. Search results are influenced by factors such as relevance, interaction, and quality, which may vary from one user to another. These limitations highlight the need for exercising caution when generalizing the findings. This study is also limited by the fact that a single evaluator assessed the videos, which could further impact the generalizability of the findings. Hence, generalizing the findings of this cross-sectional study to the wider user population could be challenging [14].

## Conclusions

Currently, YouTube hosts a plethora of videos providing healthcare information. While the quality and dependability of YouTube videos concerning gastric Botox may be deemed moderate in general based on standard scoring methodologies, we recommend the use of videos generated by academic institutions and health information websites to educate patients.
